# Meshed Versus Sheet Skin Graft for Scrotum and Perineal Skin Loss: A Retrospective Comparative Study

**DOI:** 10.7759/cureus.18348

**Published:** 2021-09-28

**Authors:** Hatan Mortada, Tareg Alhablany, Dahna Alkahtani, Mohammed Ehsan Rashidi, Abdulla Altamimi

**Affiliations:** 1 Department of Plastic Surgery & Burn Unit, King Saud Medical City, Riyadh, SAU; 2 Division of Plastic Surgery, Department of Surgery, King Saud University Medical City, King Saud University, Riyadh, SAU; 3 Department of Plastic and Reconstructive Surgery, King Abdulaziz University Faculty of Medicine, Jeddah, SAU

**Keywords:** split-thickness skin graft, genitals, reconstructive surgery, grafting, skin, skin avulsion, skin graft

## Abstract

Background

A wide array of diseases can lead to skin defects of the male genitalia. Although reconstructive options have been debated in the literature, no study has compared the effectiveness of a meshed split-thickness skin graft (STSG) and a sheet STSG in perineal and scrotal wound coverage. In this study, we report our experience in a tertiary trauma center.

Methodology

In this retrospective study, we included cases with a skin defect of the male genitalia, for which genital reconstruction with a skin graft was performed at our hospital from December 2017 to February 2020. This study was approved by the institutional review board. The analysis was performed at 95% confidence interval using the Statistical Package for Social Science (SPSS) version 23.0 (IBM Corp., Armonk, NY, USA).

Results

A total of 27 patients were included in the study. The most common indication for genital reconstruction was Fournier’s gangrene (59.3%). In 15 (55.6%) patients, a meshed skin graft was utilized to cover the defect, whereas a sheet graft was utilized in 12 (44.4%) patients. Out of the 15 patients who underwent genital reconstruction with a meshed graft, 10 (66.6%) had complete graft take. On the other hand, out of the 12 (44.4%) patients who underwent genital reconstruction using a sheet graft, five (41.6%) had complete graft take. A statistically significant relationship was found between aesthetic and functional outcomes and the type of skin graft used. The satisfaction rate was higher among meshed skin graft recipients (86.2%) compared to sheet skin graft recipients (41.7%) (p = 0.014).

Conclusions

Based on our observational experience, we found that meshed STSG to cover male genital skin defects is safe with satisfactory cosmetic outcomes. Further prospective randomized studies are needed.

## Introduction

Numerous diseases can affect the male genitalia leading to significant dysfunction, profound skin defect, and aesthetic disfigurement [1]. These diseases are diverse and range from potentially fatal soft tissue infections (Fournier’s gangrene) to relatively less common etiologies such as trauma, burn injuries, hidradenitis suppurativa, malignancies, and lymphedema [2-4]. Fournier’s gangrene is a polymicrobial necrotizing fasciitis caused by a mixture of aerobic and anaerobic bacteria. It may involve the perineum, perianal region, and genitals. Several comorbidities have been associated with Fournier’s gangrene, with the most common being diabetes mellitus and alcohol abuse [5]. In all cases of Fournier’s gangrene, surgical intervention is required to excise the affected skin, resulting in a major skin defect [1]. There are multiple techniques to reconstruct and cover these defects, such as primary closure, local flaps, full-thickness skin grafts (FTSGs), and split-thickness skin grafts (STSGs) [6]. FTSGs are composed of an entire skin layer involving the epidermis and dermis. FTSGs provide good results in terms of skin texture, color, contraction resistance, and aesthetic outcome. However, some factors limit the use of FTSGs such as limited donor sites that provide high-quality skin and impermeability to fluids which increase the risk of graft failure. STSGs are composed of epidermis and varying portions of the dermis, ranging between 8/1,000 and 12/1,000 inches. STSGs have numerous advantages such as including the inherent ability to cover larger areas and re-harvesting after donor site healing [7]. STSGs can be placed as a sheet or with meshing. In cases where additional coverage is needed or in contaminated wounds that may collect fluids underneath, the meshing of STSG is considered an ideal method with excellent outcomes. Currently, a single method that provides good functional and physical outcomes with low morbidity and reduced hospital stay remains debatable. While studies have discussed the advantages of split and sheet-thickness skin grafts, few have compared the outcomes and effectiveness of meshed versus sheet skin grafts in perineal and scrotal reconstruction. The current study is an attempt to fill this gap in the literature and to report our experience from a tertiary trauma center in the Kingdom of Saudi Arabia.

## Materials and methods

Patient selection and study design

In this single-center retrospective study, we included all patients who underwent male genital reconstruction with a skin graft at our hospital between December 2017 and February 2020. Patients who had perineal or scrotal skin loss regardless of the etiology were included in the study. Patients’ age, comorbidities, etiology of perineal and scrotal skin loss, type of skin graft applied, site of skin loss, graft uptake, length of hospital stay, aesthetic and functional outcomes, and postoperative complications were obtained from the medical records.

Ethical consideration

All patients signed an informed consent to allow the use of their preoperative and postoperative images for publication. This study was approved by the institutional review board of King Saud Medical City, Riyadh, Saudi Arabia (Ref. No. 10/6924/IRB).

Description of surgical technique

All patients were operated on under endotracheal intubation and general anesthesia. A warming mattress was used in all patients to avoid hypothermia. Preoperatively, patients were placed in the lithotomy position. The lower abdomen, genital region, and bilateral thighs were shaved. Intravenous broad-spectrum antibiotics were administered. Debridement constituted excising devitalized tissue as well as skin edges. The viability of the testicles, spermatic cord, and the extent of penile damage was confirmed by exploration. STSG was harvested from one or bilateral thighs depending on the size of the defect. The graft was harvested using mineral oil on the thigh’s anterior aspect, and a 0.015-inch thickness Zimmer dermatome (Zimmer, Indiana, USA) was used. Subsequently, Sofra-tulle (Patheon UK Limited, Swindon, UK) dressing was applied over the graft donor site. After two STSGs were harvested from the thigh, one was kept as unmeshed while the other was meshed (1:1). The unmeshed sheet graft was sutured circumferentially around the penile shaft. Vicryl 3/0 was used to suture the graft along the base of the penile shaft subcoronal tissue. On the other hand, the meshed graft was sutured with Vicryl 3/0 around the scrotum to cover the defect. A urinary catheter was placed before applying the dressing. The Sofra-tulle dressing was applied on the recipient sites, and the penile shaft was immobilized under erectile conditions using a sponge for optimal take of the graft and to ensure less mobility. For all cases, the penile shaft was stretched at the graft application time and dressing, as wrinkles within the graft can cause poor graft take. Patients were then commenced on intravenous augmentin, ciprofloxacin, and metronidazole considering the potential for developing a polymicrobial infection. Broad-spectrum intravenous antibiotics were administered for the first 24 hours and then switched to oral augmentin for five days. Graft immobility for at least five days is the most potent factor contributing to the extent of graft take. Patients were usually discharged home after initial operative management on days six to eight.

Statistical analysis

Data were checked for errors before analysis. Data analysis was performed at 95% confidence interval using the Statistical Package for Social Science (SPSS) version 23.0 (IBM Corp., Armonk, NY, USA). Categorical variables were presented as frequencies and percentages. Continuous variables were presented as mean and standard deviation. The relationship between the type of skin graft and age and length of hospital stay were assessed by the Mann-Whitney U-test. Relationship with the type of skin graft and comorbidities, etiology, site of skin loss, outcomes, aesthetic and functional outcomes, and complications were assessed by the chi-square test. A p-value of 0.05 was used to determine statistical significance.

## Results

Demographics and etiology

**Figure 1:**
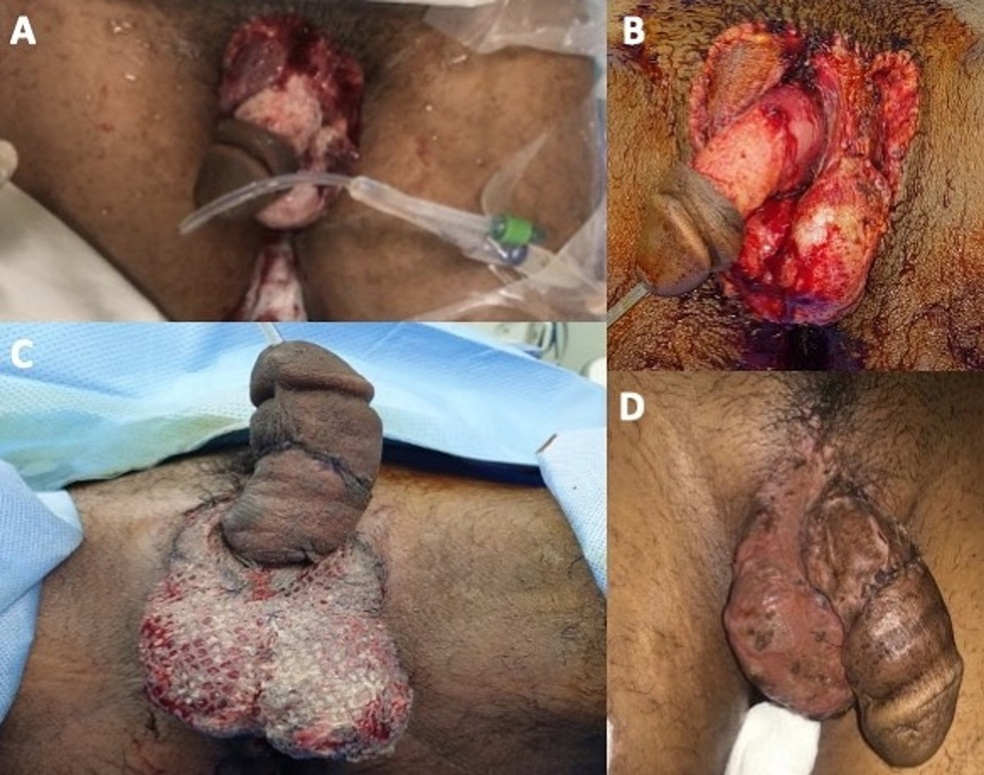
(A) A 26-year-old male with traumatic degloving injury of penile and scrotal skin. (B) After marginal debridement. (C) Three days postsurgery, first dressing. (D) Satisfying meshed STSG over the scrotum take eight weeks after the surgery. STSG: split-thickness skin graft

**Figure 2:**
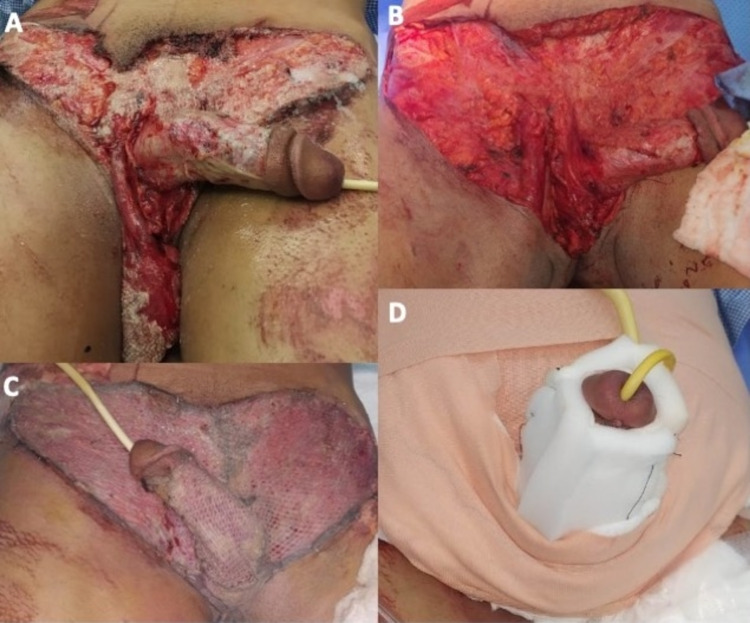
(A) A 22-year-old male with a history of traumatic degloving injury of penile, scrotal, and lower abdomen. (B) After initial debridement. (C) Immediately postoperative prior to dressing application. (D) After application fluffs and penile splint over the graft.

**Figure 3:**
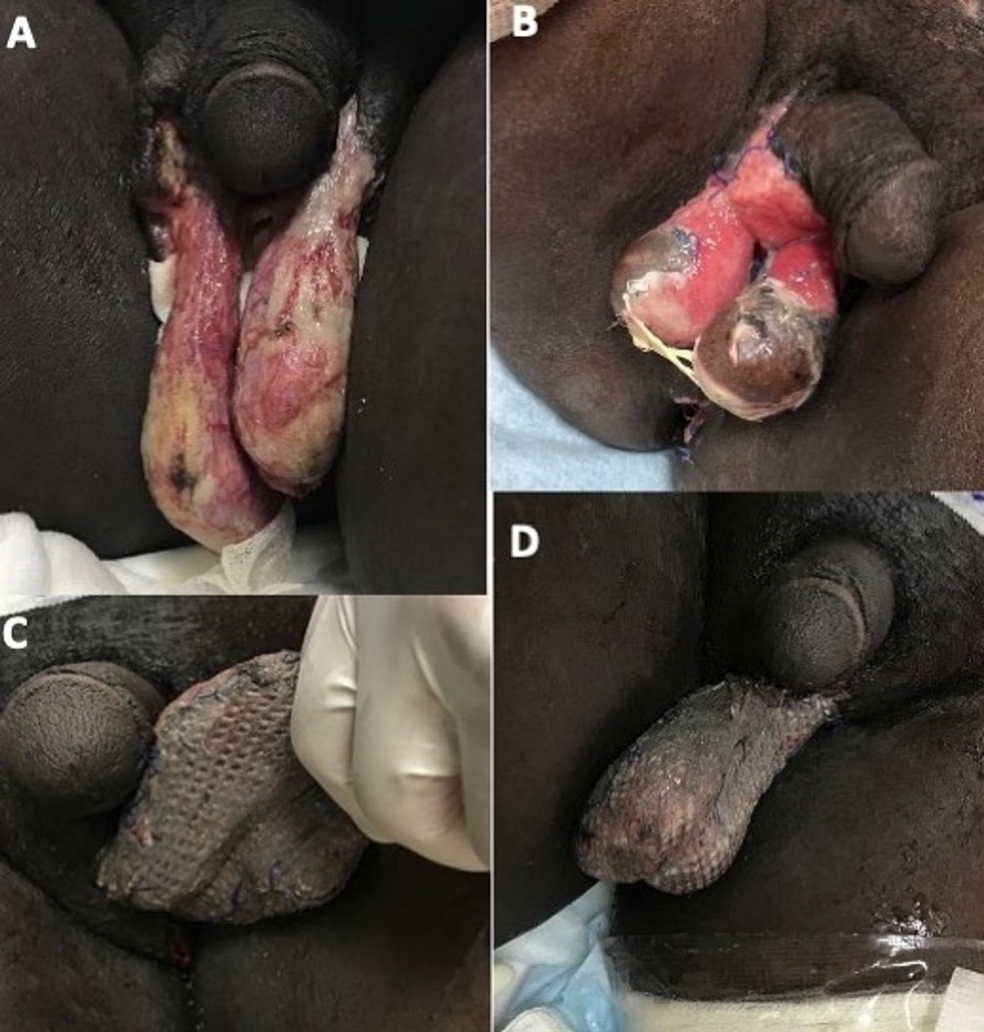
(A) A 50-year-old male with a history of diabetes mellitus and Fournier’s gangrene of scrotal skin. (B) Failure of first attempted skin graft application due to infection. (C, D) Successful second attempt meshed STSG application one week postsurgery. STSG: split-thickness skin graft

A total of 27 males were retrospectively included in the study who suffered scrotal and/or perineal skin loss. The mean age was 35.26 ± 13.35 years. Of the 27 patients, 18 (66.6%) had comorbidities, and the most common comorbidity was diabetes mellitus in nine (33.3%) patients. In total, three (11.1%) patients were obese. Others had arterial hypertension, autoimmune disease, and hyperlipidemia. The most common cause of scrotal and perineal skin loss was Fournier’s gangrene observed in 16 (59.3%) patients. The most common site of skin loss was the “penis, scrotum, and perineum” (40.7%). Table 1 summarizes patients included in the study, and Figures 1-3 show the images of some of the patients. A successful outcome of the skin graft was seen in 15 (55.6%) patients, and satisfying aesthetic and functional outcomes were seen in 18 (66.7%) patients. Complications were seen in 12 (44.44%) patients. The most common complication was “complete graft loss” in eight (29.6%) patients (Tables 2, 3). In 15 (55.6%) patients, a meshed skin graft was used, while in 12 (44.4%) patients a sheet graft was used. The choice of using a meshed or a sheet graft was individual consultant preference.

**Table 1:** Patients included in the study. STSG: split-thickness skin graft; HTN: hypertension

Case	Age	Comorbidities	Etiology	Type of STSG applied	Site of skin loss	Postoperative outcome	Aesthetic and functional outcome of graft
1	25	Autoimmune disorder	Traumatic degloving injury	Meshed	Penis, scrotum, and perineum	Success	Satisfying
2	40	Diabetes mellitus	Fournier’s gangrene	Sheet	Complete skin loss over the penis	Failure	Unsatisfying
3	36	HTN, diabetes mellitus	Fournier’s gangrene	Meshed	Complete skin loss over the penis	Success	Satisfying
4	19	None	Lymphedema	Meshed	Penis, scrotum, and perineum	Success	Satisfying
5	38	None	Hidradenitis suppurativa	Meshed	Isolated scrotal skin loss	Success	Satisfying
6	28	None	Traumatic degloving injury	Meshed	Complete skin loss over the penis	Success	Satisfying
7	60	Diabetes mellitus	Fournier’s gangrene	Sheet	Complete skin loss over the penis	Failure	Unsatisfying
8	50	Diabetes mellitus	Fournier’s gangrene	Meshed	Isolated scrotal skin loss	Success	Satisfying
9	20	Obesity	Fournier’s gangrene	Sheet	Penis, scrotum, and perineum	Failure	Unsatisfying
10	50	Diabetes mellitus	Fournier’s gangrene	Meshed	Isolated scrotal skin loss	Success	Satisfying
11	26	None	Traumatic degloving injury	Sheet	Complete skin loss over the penis	Failure	Unsatisfying
12	29	Obesity	Fournier’s gangrene	Meshed	Isolated scrotal skin loss	Success	Satisfying
13	22	None	Traumatic degloving injury	Meshed	Complete skin loss over the penis	Partial uptake	Satisfying
14	56	Diabetes mellitus	Fournier’s gangrene	Meshed	Penis, scrotum, and perineum	Partial uptake	Satisfying
15	61	HTN, diabetes mellitus	Fournier’s gangrene	Meshed	Isolated scrotal skin loss	Partial uptake	Unsatisfying
16	39	HTN	Fournier’s gangrene	Sheet	Penis, scrotum, and perineum	Partial uptake	Satisfying
17	22	None	Hidradenitis suppurativa	Sheet	Penis, scrotum, and perineum	Partial uptake	Satisfying
18	18	None	Hidradenitis suppurativa	Meshed	Complete skin loss over the penis	Success	Satisfying
19	55	Diabetes mellitus	Fournier’s gangrene	Sheet	Isolated scrotal skin loss	Failure	Unsatisfying
20	39	Diabetes mellitus	Fournier’s gangrene	Sheet	Penis, scrotum, and perineum	Success	Satisfying
21	24	None	Fournier’s gangrene	Meshed	Partial skin loss over penis	Failure	Unsatisfying
22	32	Hyperlipidemia	Fournier’s gangrene	Sheet	Penis, scrotum, and perineum	Success	Satisfying
23	26	Autoimmune disorder	Burn	Sheet	Penis, scrotum, and perineum	Success	Satisfying
24	35	Diabetes mellitus	Fournier’s gangrene	Meshed	Partial skin loss over the penis	Success	Satisfying
25	34	Obesity	Lymphedema	Sheet	Partial skin loss over the penis	Success	Unsatisfying
26	47	Diabetes mellitus	Fournier’s gangrene	Meshed	Penis, scrotum, and perineum	Success	Satisfying
27	18	None	Traumatic degloving injury	Sheet	Penis, scrotum, and perineum	Success	Unsatisfying

**Table 2:** Age and length of hospital stay of patients.

Variables	Mean	Standard deviation	Median
Age in years	35.26	13.346	34
Length of hospital stay (days)	4.7407	1.81007	4

**Table 3:** Categorical variables.

Variables	N	%
Comorbidities		
Yes	9	33.3
No	18	66.7
Etiology		
Fournier’s gangrene	16	59.3
Burn	4	14.8
Hidradenitis suppurativa	3	11.1
Lymphedema	2	7.4
Traumatic degloving injury	1	3.7
Type of skin graft		
Meshed	15	55.6
Sheet	12	44.4
Site of skin loss		
Penis, scrotum, and perineum	11	40.7
Complete skin loss over the penis	7	25.9
Isolated scrotal skin loss	6	22.2
Partial skin loss over penis	3	11.1
Outcome of skin graft		
Success	15	55.6
Failure	6	22.2
Partially uptake	6	22.2
Aesthetic and functional outcome		
Satisfying	18	66.7
Unsatisfying	9	33.3
Complications		
None	16	59.3
Complete graft loss	8	29.6
Hematoma	1	3.7
Infection	1	3.7
Seroma	1	3.7

Meshed split-thickness skin graft

Out of 15 patients in whom meshed graft was used, 10 (66.6%) had complete graft take. Overall, three (20%) patients had graft loss mounting to 10%, one had graft loss of more than 30% and one had complete graft loss. These cases were managed conservatively and healed by secondary intention. None of the patients in this group required a second surgery. In total, nine (60%) patients were discharged after the first postoperative dressing on days three to four. The remaining six patients were discharged after the second postoperative dressing on days five to six.

Sheet skin graft

Out of 12 (44.4%) patients in whom sheet graft was used, only 5 (41.6%) had complete graft take. Two patients had graft loss of more than 25%, while five had more than 50% graft loss. In these five patients in whom 50% of the graft was lost, a second surgery was done and a sheet graft was applied; the remaining two patients in whom 25% graft was lost were managed conservatively. Five patients in this group were discharged on day five after the second dressing, two patients on day seven, and five patients in whom the second surgery was done were discharged on day ten after the first surgery. The average length of stay in the meshed group was 4.4 days (range: 3-7 days), while in the sheet group, it was 5.2 days (range: 3-9 days). Both groups were followed up in the outpatient department and showed similar aesthetic and functional outcomes.

No statistically significant relationship was found between the type of skin graft and the age of the patients (p = 0.903). In addition, no statistically significant relationship was found between the type of skin graft and the length of hospital stay (p = 0.354) (Table 4).

**Table 4:** Relationship between the type of skin graft and age and length of hospital stay.

Variables	Meshed	Sheet	P-value
Age	35.87 ± 13.964	34.50 ± 13.104	0.903
Length of hospital stay	4.4000 ± 1.50238	5.1667 ± 2.12489	0.354

The presence of comorbidities, etiology of skin loss, site of skin loss, outcome of the surgery, and presence of complications were not statistically significantly related to the type of skin graft (all p-values > 0.050). The only statistically significant relationship was between aesthetic and functional outcomes and the type of skin graft used. The satisfaction rate was higher among meshed skin graft recipients (86.2%) compared to sheet skin graft recipients (41.7%) (p-value = 0.014) (Table 5).

**Table 5:** Relationship between categorical variables.

Variables	Meshed (%)	Sheet (%)	P-value
Comorbidities			0.411
Yes	60	75	
No	40	25	
Etiology			0.655
Burn	20	8.3	
Fournier’s gangrene	60	58.3	
Hardenites suppurativa	13.3	8.3	
Lymphedema	6.7	8.3	
Traumatic degloving injury	0	8.3	
Site of skin loss			0.299
Complete skin loss over the penis	26.7	25	
Isolated scrotal skin loss	33.3	8.3	
Partial skin loss over the penis	13.3	8.3	
Penis, scrotum, and perineum	26.7	58.3	
Outcome			0.094
Failure	6.7	41.7	
Partial uptake	26.7	16.7	
Success	66.7	41.7	
Aesthetic and functional outcome			0.014
Satisfying	86.7	41.7	
Unsatisfying	13.3	58.3	
Complications			0.096
Yes	73.3	41.7	
No	26.7	58.3	

## Discussion

Perineal wound defects are challenging to manage. The main goal of reconstruction is wound coverage, preservation of voiding, sexual function, and a good cosmetic outcome [1,8]. The wide range of reconstructive options includes primary closure, local flaps, FTSG, and STSG. Local flaps such as the groin flap and the lateral thigh flap are commonly used procedures as they provide a good aesthetic result. Radial forearm free flap, anterolateral thigh flap, and latissimus dorsi flap are other distant flaps used for such defects. Although these flaps are aesthetically promising, they are limited by donor-site comorbidities, length of hospital stay, flap failure, and cost-effectiveness. Considering these limitations, reconstructive surgeons now prefer skin grafts due to their convenient technique and good outcome; however, the procedure can be difficult in perineal wounds because of the location [7-9].

Although FTSGs are thought to provide a superior aesthetic result, there are some disadvantages such as limited donor sites that provide high-quality skin and donor site comorbidity. STSGs have several advantages over FTSGs including good graft take in cases of wound contamination such as trauma, avulsion injuries, burns, and hidradenitis [9,10]. Skin grafting in the perineal area has a risk of graft failure due to bacterial infection, difficulty in the stabilization of the graft due to the mobility of genitalia, and fluid collection in the form of blood, serous, and purulent discharge. However, graft meshing helps the graft to contour well with the wound’s granulated bed and to facilitate fluid drainage [8]. To prevent highly infective discharge, surgeons prefer negative-pressure wound therapy (NPWT) dressing before and after the surgery [10-13]. NPWT dressing in these areas is a challenge due to the difficulty in creating airtight dressing due to uneven surface and mobility. Alwaal et al. [1] demonstrated the successful utilization of a meshed STSG for scrotal reconstruction. Chen et al. [12] also reported the use of FTSG in managing Fournier’s gangrene with large tissue defects. Similar to previous reports, we report the successful experience of meshed STSGs for perineal reconstruction due to diverse etiologies (Fournier’s gangrene, burn, hidradenitis suppurativa, lymphedema, and traumatic degloving injury). In our study, meshed grafts survived to a greater extent compared to sheet grafts. On the other hand, sheet grafts are used to cover the penile shaft to avoid contraction [14] and unsatisfactory aesthetic results. Black et al. [6] reported the use of meshed STSGs for penile tissue loss. This is consistent with a study conducted by Alkahtani et al. [15] who applied an unmeshed sheet graft over the penile shaft skin defect, along with a meshed STSG to cover the scrotal defect. In our study, we observed a difference between sheet and meshed STSGs in terms of aesthetic and functional outcomes. Furthermore, there was a statistically significant relationship between aesthetic and functional outcomes and the type of skin graft used. In addition, the satisfaction rate was higher among meshed skin graft recipients (86.2%) compared to sheet skin graft recipients (41.7%) (p-value = 0.014). Further prospective studies are needed to confirm these findings.

Limitations

This study was limited by the small number of patients. This might be attributed to the low incidence of perineal and scrotal skin losses. The assessment of functional outcome was subjective, and the International Index of Erectile Function Questionnaire was not used. To date, there is no standard method of assessment for graft take; thus, our graft take assessment was done based on the surgeon’s clinical judgment and expertise. There is an inherent need for further clinical studies to include a larger population size, for improved validity, as well as to utilize validated tools in the assessment of function, clinical, and cosmetic outcomes. In addition, prospective cohort studies are needed to confirm the results of this study.

## Conclusions

In the context of male genital skin defects, a single-step reconstructive procedure using a meshed skin graft to cover the skin defect has proven to be the superior option compared to a sheet graft for achieving good aesthetic and functional outcomes. Based on our experience, a meshed skin graft to cover male genital skin defects is safe and demonstrates satisfactory cosmetic outcomes. Further, we propose making male genital reconstruction using a meshed graft the standard of care offered as initial surgical management. However, further prospective studies are needed to confirm these findings.
